# Astrocytes of the eye and optic nerve: heterogeneous populations with unique functions mediate axonal resilience and vulnerability to glaucoma

**DOI:** 10.3389/fopht.2023.1217137

**Published:** 2023-07-21

**Authors:** Paul F. Cullen, Daniel Sun

**Affiliations:** Department of Ophthalmology, Schepens Eye Research Institute of Massachusetts Eye and Ear, Harvard Medical School, Boston, MA, United States

**Keywords:** optic nerve head astrocytes, glaucoma, reactive astrocytes, neurodegeneration, astrocyte heterogeneity, astrocyte metabolism, mitochondria, gap junctions

## Abstract

The role of glia, particularly astrocytes, in mediating the central nervous system’s response to injury and neurodegenerative disease is an increasingly well studied topic. These cells perform myriad support functions under physiological conditions but undergo behavioral changes – collectively referred to as ‘reactivity’ – in response to the disruption of neuronal homeostasis from insults, including glaucoma. However, much remains unknown about how reactivity alters disease progression – both beneficially and detrimentally – and whether these changes can be therapeutically modulated to improve outcomes. Historically, the heterogeneity of astrocyte behavior has been insufficiently addressed under both physiological and pathological conditions, resulting in a fragmented and often contradictory understanding of their contributions to health and disease. Thanks to increased focus in recent years, we now know this heterogeneity encompasses both intrinsic variation in physiological function and insult-specific changes that vary between pathologies. Although previous studies demonstrate astrocytic alterations in glaucoma, both in human disease and animal models, generally these findings do not conclusively link astrocytes to causative roles in neuroprotection or degeneration, rather than a subsequent response. Efforts to bolster our understanding by drawing on knowledge of brain astrocytes has been constrained by the primacy in the literature of findings from peri-synaptic ‘gray matter’ astrocytes, whereas much early degeneration in glaucoma occurs in axonal regions populated by fibrous ‘white matter’ astrocytes. However, by focusing on findings from astrocytes of the anterior visual pathway – those of the retina, unmyelinated optic nerve head, and myelinated optic nerve regions – we aim to highlight aspects of their behavior that may contribute to axonal vulnerability and glaucoma progression, including roles in mitochondrial turnover and energy provisioning. Furthermore, we posit that astrocytes of the retina, optic nerve head and myelinated optic nerve, although sharing developmental origins and linked by a network of gap junctions, may be best understood as distinct populations residing in markedly different niches with accompanying functional specializations. A closer investigation of their behavioral repertoires may elucidate not only their role in glaucoma, but also mechanisms to induce protective behaviors that can impede the progressive axonal damage and retinal ganglion cell death that drive vision loss in this devastating condition.

## Introduction

Glaucoma, a complex disease characterized by the progressive dysfunction and permanent loss of retinal ganglion cells (RGCs), is the leading cause of irreversible blindness, affecting nearly 80 million individuals worldwide (1, 2). Its prevalence places it among the most common neurodegenerative diseases, and the visual impairment it causes makes it a major health and economic burden (3–5). While the broad contours of glaucomatous progression are straightforward – the death of retinal ganglion cells and a resultant loss in visual field – the etiology of the disease remains incompletely understood, particularly for primary open-angle glaucoma, the most common variant (6, 7). Age and elevated intraocular pressure (IOP) are major risk factors, while genetics also play a role, but current management strategies are largely limited to the lowering of IOP (1, 2). However, the relationship between IOP and progression is not absolute: many individuals with ocular hypertension do not develop glaucoma, some individuals with IOPs in the normal range do, and some percentage of treated individuals nevertheless continue losing visual acuity (2, 8). Despite the demonstrated need for directly neuroprotective therapies to halt the loss of RGCs and preserve vision, either as an adjunct to IOP control or in place of it, such an approach remains out of reach, in part because of our incomplete understanding of the pathophysiology of glaucoma. Nonetheless, new mechanistic insights into the progression of glaucoma may also shed light on other age-related neurodegenerative diseases, moving the field closer to viable treatments and preventative strategies.

Astrocytes, a type of glial support cell found throughout the central nervous system (CNS), are of particular interest in the pathophysiology of glaucoma because they constitute the main cellular component of the optic nerve head (ONH), a key site of early pathological events (2, 9, 10). During glaucoma they transition to a reactive state, a process that drives changes in their morphology, function and gene expression that can influence their support for RGCs, alter the tissue environment and influence disease outcome (11–14). Despite growing awareness of their relevance, both in glaucoma and neurodegenerative diseases more broadly, the behavior of astrocytes in glaucoma remains poorly understood. This is linked more broadly to an incomplete understanding of white matter astrocytes, the type that populates both the optic nerve and retina, in terms of heterogeneity and behavior under both physiological and pathological conditions. Although the heterogeneity of astrocytes has recently expanded as a research topic, much of this work has been done in gray matter astrocytes of the brain; the extent of differences between white matter and gray matter astrocytes is still emerging, while heterogeneity within and between white matter astrocyte populations remains largely unknown. A more in-depth understanding of the characteristics of white matter astrocytes is required if we are to better learn their unique functions in disease.

## Astrocyte function and heterogeneity

The most abundant glial cell type in the central nervous system, astrocytes were once thought of as passive structural support to neurons (15). While astrocytes do play key structural roles through both their influence on the extracellular matrix (ECM) and their own intrinsic biomechanical properties, they are now recognized as performing a broad array of functions essential to supporting neurons and maintaining a healthy CNS (16–18). Individually, astrocytes contact blood vessels and neuronal structures, rapidly shuttling energy substrates to active neurons while syphoning away excess ions and neurotransmitters released during neuronal signaling (19–23). However, astrocytes also form extensive networks, linked by gap junctions, that facilitate large scale spatial buffering of factors within the neural parenchyma as well as the coordination of astrocyte behavior (24, 25). Astrocytes additionally function as secretory cells, releasing neurotrophic and synaptogenic factors, lipids with beneficial or detrimental effects on neuronal survival, and chemokines that mediate immune response by signaling microglia and peripheral immune cells (26–31). Beyond these core functions are a wide variety of specialized behaviors that are indicative of the still-emerging extent of astrocyte heterogeneity (32–35).

From the early days of modern neuroscience, a degree of astrocyte heterogeneity has been recognized, with researchers categorizing astrocytes as either protoplasmic or fibrous, based on morphology (36). These distinctions largely align with anatomical localization – protoplasmic astrocytes generally populate synaptically dense gray matter regions, whereas fibrous astrocytes typically reside in myelinated white matter tracts – and correlate to functional specialization at the broadest level (37). Gray matter astrocytes, for example, contact large numbers of synapses, where they are involved in supporting and modulating synaptic function, ranging from the uptake of excess glutamate to altering signaling sensitivity to the preservation or elimination of individual synapses; the extent of their participation has given rise to the concept of the tripartite synapse, in which astrocytes participate in synaptic regulation alongside neuronal axons and dendrites (28, 38, 39). Conversely, astrocytes in white matter contact neurons along their axons, modulate oligodendrocyte function to preserve unmyelinated contact points at the Nodes of Ranvier, and incorporate oligodendrocytes within their gap junction connected networks (40–43). Historically gray matter astrocytes have been more heavily investigated – despite the vulnerability of white matter regions such as the optic nerve tract in glaucoma, multiple sclerosis, and other debilitating neurological conditions – and eventually became the basis of ‘normal’ astrocyte function in much of the literature (33, 44, 45). An exception to this trend has been the investigation of energy supplementation of neurons by astrocytes, where much foundational work was done with optic nerves for practical reasons (23, 46).

More recently, however, heterogeneity within these two types has emerged as well, and a number of regional populations of astrocytes have demonstrated specialized functions, many involving physiological and behavioral activities that were previously thought to be mediated by neurons. Among gray matter astrocytes, those in the cortex have been shown to participate directly in neurovascular coupling (47), while a population at the ventral surface of the medulla oblongata detects pH changes in the blood and triggers the neuronal response that drives reflex breathing (48), and those of suprachiasmatic nucleus are able to drive circadian rhythm cycles independently of neuronal time-keeping (49). Nor are region specific populations the limit of astrocyte heterogeneity, as studies within regions have identified sub-populations with morphological and transcriptional variation: within the cortex, morphological studies identified four sub-populations of protoplasmic astrocytes, a finding replicated with single cell transcriptomic analysis (50, 51). Here too is our current understanding largely dominated by findings on gray matter astrocytes, despite evidence of morphological and functional heterogeneity in white matter tracts such as the optic nerve (52–55). Two exceptions documented in the optic nerve – the involvement of astrocytes in axonal mitochondria turnover and non-cell autonomous provisioning of energy substrates to axons – are of particular interest in the context of glaucoma and addressed in detail later in this review (56, 57).

## Astrocyte reactivity

In addition to inter- and intra- regional heterogeneity of astrocytes under physiological conditions, the changes undergone by astrocytes in response to disruption of homeostasis compound the complexity of the matter still further. When the central nervous system suffers insult – such as physical trauma, infection by pathogens, or ischemia – astrocytes respond by undergoing a transition to a reactive state (sometimes referred to as gliosis), in which their behavior is altered – acutely or chronically – to address the altered conditions they are exposed to (58–60). In addition to their response to relatively straightforward exogenous insults, reactive astrocytes are a major area of research in chronic neurodegenerative conditions, including glaucoma (7, 61–64). Astrocyte reactivity is closely linked to neuroinflammation, a broader inflammation-like state that occurs in central nervous system injury and neurodegeneration, which is characterized by the response of astrocytes, microglia, and other resident cell types, alongside contributions from the peripheral immune system (12, 65, 66). As with conventional inflammation, neuroinflammation is an essential response to insult that nonetheless can drive deleterious outcomes, particularly when it persists in a chronic state (67, 68). However, as with other elements of astrocyte behavior, the heterogeneity of these responses is only recently coming into focus (59, 60, 69).

Historically, reactivity has been associated with its most severe manifestations, including astrocyte hypertrophy, proliferation, and scar formation, and thought to be the primary barrier to regeneration in the central nervous system of mammals (70, 71). Subsequent work demonstrated reactivity to be a graded phenomenon, with many of the above responses constrained to cases of particularly grievous injury (16). Still more recent research demonstrates an essential role for reactivity in preserving nervous tissue in the face of damaging stimuli, revealing a complex behavioral response that can also include neuroprotective functions (72, 73). The astrocyte-specific deletion of transcription factor STAT3, for example, diminishes the reactive capacity of astrocytes and leads to a worsening of visual function in a mouse model of glaucoma (74). A high-profile effort has been made in recent years to better understand reactive astrocytes by classifying them as detrimental (Neurotoxic/A1) or beneficial (Neuroprotective/A2) (75, 76). However, a recent consensus statement by a number of major figures in the field of astrocyte research makes it clear that this duality understates the degree of relevant heterogeneity (69). Rather, the reactive response of astrocytes depends on a multitude of factors, including the site, type, duration, and severity of insult, as well as the preexisting state of the astrocytes in question; indeed, many subsequent studies have found overlapping expression of A1 and A2 markers in reactive astrocytes from human tissue and in a variety of mouse models (77–79). Finally, further compounding this complexity is the role of aging, which appears to drive a pseudo-reactive state in astrocytes that potentiates reactive changes and may contribute to the increased vulnerability of aged individuals to neurodegenerative diseases (80–82).

This complex heterogeneity suggests that reactivity is a loose constellation of interrelated behaviors, each of which may or may not be present in a given population of reactive astrocytes. Crucially, it also suggests that although a broad understanding of astrocyte behavior may hint at their role in glaucoma, the behavior of astrocytes elsewhere in the CNS – whether under physiological or pathological conditions – may not be predictive of those attending the axons of RGCs in the optic nerve and retina.

Therefore, the remainder of this review will focus on the astrocyte populations of the anterior visual pathway – the retina, the optic nerve head, and myelinated optic nerve – and recent notable work in the field, including a transcriptional study by our group that corroborates previous functional and morphological studies suggesting that the astrocytes of the optic nerve head form a distinct population from those of the myelinated optic nerve.

## Zones of the retinal ganglion cell axon and attendant white matter astrocyte populations

Retinal ganglion cell axons, the most structurally and metabolically (45) vulnerable component of the visual pathway, traverse three distinct ‘zones’ (9, 14) after emerging from the somas of RGCs. These regions – the retinal nerve fiber layer, optic nerve head, and myelinated optic nerve – expose the axons to widely divergent environments. In the retina RGC axons are unmyelinated, increasing their metabolic vulnerability, but the nerve fiber layer possesses a dense capillary bed that may offset the lack of myelin (14). Conversely, at the optic nerve head, these still unmyelinated axons undergo a sharp turn and pass through a pressure differential across the lamina, exposing them to both mechanical and metabolic stressors; perhaps as a consequence, despite extensive vascularization the ONH is a major site of early damage in glaucoma (9, 14, 83, 84). The myelinated portion of the optic nerve, the largest axon section by far, emerges posteriorly from the ONH; like the retina, it is not thought to be an early site of glaucomatous damage.

Throughout these three regions, RGC axons are supported by fibrous astrocytes that emerge from precursors in the optic nerve during development (85–88). Despite common developmental origins, astrocytes residing in each region show evidence of specialized adaptations that may be indicative of distinct populations. The astrocytes of the optic nerve, in particular, have historically been considered as a homogeneous population, although the specialized anatomy of the optic nerve head and its role as a key site of early glaucomatous damage later gave rise to a specific interest in the functions of astrocytes there. Recent functional studies have highlighted specialized roles for these astrocytes that may be unique, and a transcriptional study of astrocytes in the optic nerve published by our group highlights that these astrocytes may be best understood as a distinct population from those of the myelinated optic nerve (55, 89, 90). Intriguingly, other recent work suggests that these populations are nevertheless extensively networked across anatomically large distances by gap junctions, facilitating coordination of non-cell autonomous support functions at a previously unappreciated scale (57). A greater understanding of both the heterogeneity between these populations and their capacity for coordinated behavior may shed new light on the mechanisms of glaucomatous vulnerability and new avenues of neuroprotection.

## Retinal astrocytes

In the furthest anterior region of the visual pathway, a distinct population of fibrous astrocytes tiles the vitreoretinal surface in many mammals – including humans - and is required for retinal vascularization during development (91–94). These retinal astrocytes interweave RGC axons in the nerve fiber layer and contact the superficial retinal vasculature, but their roles in neurovascular coupling and ionic buffering remain inconclusive (21, 95, 96). In humans and other primates, these astrocytes fall into two morphological categories, with more ‘stereotypical’ stellate astrocytes contacting the vasculature in the ganglion cell layer of the retina, while astrocytes with elongated processes that run parallel to axons are found in the nerve fiber layer (97, 98). The extent of heterogeneity and specialization of these two variants is largely unknown; recent single-cell RNA sequencing experiments with human tissue have generally been able to distinguish radial Müller glia from astrocytes, but not subtypes within these categories (99, 100). While at least one study found greater variation, identifying three distinct glial clusters, it could not conclusively distinguish which may have represented Müller glia and which may have been astrocytic (101). Regardless, due to the nature of the techniques involved, none of these studies were able to link transcriptional differences to morphological variation, leaving the significance of the morphological categories uncertain.

Rats and mice, which act as the basis of many rodent models of glaucoma, do not possess such obvious morphological variation among retinal astrocytes, but some studies have assessed a greater variety in morphology via intracellular tracer dye injection (102), while others have noted trends such as the association of larger astrocytes with retinal veins rather than arteries (103). Generally, the sparseness of these astrocytes, which make up approximately 0.1% of retinal cells, renders their study a particular challenge, which is exacerbated due to the transcriptional similarity of Müller glia that outnumber retinal astrocytes by at least an order of magnitude (104, 105). However, retinal astrocytes are known to form gap junction coupled networks with directional properties, including unidirectional flow into Müller glia, which are not directly coupled to each other (106). Retinal astrocytes exhibit indicators of reactivity – such as upregulation of GFAP and remodeling – in both human glaucoma and animal models, but the extent of their causal role in disease progression remains unknown (107–109). Curiously, this reactivity has been repeatedly demonstrated to propagate from injured retina to uninjured contralateral eye in animal models, through a mechanism that has long been unclear (110, 111). A potential mechanism for this spread has been recently elucidated through a study on resource sharing across these anatomical distances, highlighting a potential neuroprotective role behind this previously puzzling function (57). Cx43, the gap junction protein that is essential for this phenomenon, is also upregulated in retinal astrocytes in human glaucoma (112).

## Optic nerve head astrocytes

Although longitudinally the shortest of these three regions, the optic nerve head is the most structurally complex and the one most directly implicated in early glaucomatous progression (9). Formed by fusion of the optic stalk and retina during development, the ONH is a transition zone between these tissues that contains elements of both, consisting of pre-laminar areas where the retina transitions to optic nerve, the lamina itself, and the posterior myelin transition zone, which is more pronounced in rodents (9, 14, 113). Although the lamina cribrosa of humans and other large mammals is more elaborate than the glial lamina of the mouse, the structures serve the same function, forming a tight seal between the positive pressure zone inside the eye and the optic nerve, isolating the retina from exposure to cerebral spinal fluid while guiding and ensheathing RGC axons as they exit the eye (14, 114, 115). Axons in this region are unmyelinated and characterized by elevated mitochondrial density, which may reflect increased metabolic requirements driven by both the lack of myelination and the challenge of axonal transport across a pressure gradient; the complex vascularization at this site may be similarly indicative of high metabolic needs (9, 14, 116).

Optic nerve head astrocytes play a major role in the structure of the lamina: in humans, astrocytes line the collagenous beams that form the mesh-like structure and insulate axons from connective tissue and vasculature, while in mice these collagen fibers are absent and astrocytic processes are instead responsible for the structural seal (9). In keeping with their structural role, these astrocytes express high levels of the intermediate filaments GFAP, vimentin (117), and nestin (118), and are consistently oriented transversely to the path of axons, adopting in mouse a distinct morphology similar to a “catcher’s mitt” in baseball (53). Uniquely among astrocytes, those at the lamina largely lack aquaporins, the specialized water channels that are so ubiquitously expressed on astrocytes elsewhere (54, 119). This absence has been observed in both humans and all investigated mammalian models, indicating that this absence is a highly conserved trait; it has been speculated that this may be a requisite for maintaining additional rigidity in these astrocytes (89).

Our recent transcriptional study of astrocytes under physiological conditions in the normo-tensive mouse optic nerve highlights a number of key differences between those of the ONH and the myelinated optic nerve proper, bolstering the idea that this a distinct population (90). Unsurprisingly, given the role of ONH astrocytes in remodeling the extracellular matrix during glaucoma, under physiological conditions we found that pathways related to ECM maintenance were highly enriched in this population relative to those of the myelinated optic nerve and corpus callosum (13). Intriguingly, we also observed clear metabolic differences between myelinated and unmyelinated optic nerve, with the astrocytes of the ONH expressing elevated levels of genes related to mitochondrial protein translation and oxidative phosphorylation. This may imply that these astrocytes are able to utilize the dense vascularization of the ONH by relying on the more energetically favorable breakdown of glucose via oxidative phosphorylation.

However, this comes with potential drawbacks, which may contribute to the heightened vulnerability of this region to glaucomatous damage. Elevated levels of oxidative phosphorylation result in increased production of reactive oxygen species (ROS), a source of oxidative stress and potential contributor to glaucoma (12, 120). Conversely, astrocytes provide antioxidant support in the central nervous system through glutathione production, the peroxiredoxin system, and other mechanisms, and our transcriptional results suggest that this is the case at the ONH as well, with high levels of glutathione transferase and peroxidase transcription implying that this population of astrocytes acts to ameliorate the effects of local production of ROS (90, 121, 122). Secondly, the enrichment of oxidative phosphorylation related genes was accompanied by indicators of lower levels of glycolysis, an alternative mechanism by which astrocytes generate energy in the brain (123). Although glycolysis generates less energy than oxidative phosphorylation, it is also tied to the production of lactate, which astrocytes use to provision neurons with energy during periods of elevated energy consumption (20). Our transcriptional comparison of ONH astrocytes and those of the corpus collosum suggests that less glycogen storage may be occurring at the ONH. Given that astrocytic glycogen forms the primary energy reserve in the CNS, this may exacerbate metabolic vulnerability; however, a recent study has shown long-range energy provisioning through the interconnected astrocyte network of the optic nerve that may offset – at least in the short term – this vulnerability (23, 57). Nonetheless, it is reasonable to hypothesize that even a modest loss of function regarding astrocytic ROS-amelioration or energy provisioning, due to aging or other contributing factors, could precipitate axonal damage in this metabolically demanding and vulnerable region.

Astrocytes of the ONH are also thought to directly sense and respond to mechanical pressure, which may influence their contributions to remodeling during glaucoma, although many of these changes may be driven in part by damage to RGC axons, such as the ‘gap-plugging’ response brought on by axonal loss (124, 125). In addition to upregulating GFAP and the gap junction protein Cx43 (112), reactive ONH astrocytes in human glaucoma and animal models extensively modify the extracellular matrix at the optic nerve head (11, 13, 126). Finally, while neuroinflammation in glaucoma includes, and is in part mediated by, astrocyte reactivity at the optic nerve head – including a complex milieu of behaviors such as antioxidant activity, nitric oxide production, and changing levels of reactive oxygen species formation – the topic also incorporates complex and incompletely elucidated interactions with the activities of additional cell types such as microglia (120, 127, 128). In order to retain the focus on astrocyte-specific functions we have largely bypassed the broader discussion of neuroinflammation in glaucoma, but the topic has been well reviewed elsewhere (7, 12).

## Myelinated optic nerve astrocytes

Directly posterior to the lamina, the transition to myelinated optic nerve begins. In mice a pronounced myelin transition zone can be observed, although this may be largely the function of differences in scale and lamina extent between mice and primates. Astrocytes in this liminal region play a major role in the turnover of axonal mitochondria, discussed later. The myelinated portion of the optic nerve extends posteriorly through retrobulbar space to the lateral geniculate nucleus, superior colliculus, and other regions to which RGCs synaptically project (9). Unlike in the retina and optic nerve head, this region is populated by oligodendrocytes that myelinate the axons, insulating them electrically and protecting them from noxious stimuli (41). Due to its straightforward anatomy and relative accessibility, the myelinated optic nerve is a classic model for studying the behavior of axons and glia in white matter (41, 129, 130). In addition to astrocytes and oligodendrocytes, this region is populated by NG2 glia, which have been previously described as oligodendrocyte precursors but may also play specific roles in neuroinflammation, and by a significantly higher density of microglia than is found at the optic nerve head (131).

Astrocytes of the myelinated optic nerve make less extensive contact with axons than those in unmyelinated regions, primarily via gaps at Nodes of Ranvier, where they take up the potassium ions released by axonal signaling and shunt them to areas of lower concentration or to the vasculature (21, 132–134). They are also responsible for providing energy substrates to axons from stored glycogen during decreased glucose availability or heightened axonal need; astrocytic energy provisioning is well studied in this portion of the optic nerve and is the focus of a more detailed section below (46, 129). Astrocytes of this region, like those of the retina and ONH, are extensively coupled by gap junctions (135), but also interact with oligodendrocytes and myelin to modulate the extent of myelination and preserve the accessibility of nodes of Ranvier (42).

## Axonal transport and mitochondrial turnover

In keeping with the metabolically demanding nature of the central nervous system, axons consume large quantities of energy, both to maintain the ionic gradients required for action potentials and for pre-synaptic elements of vesicular signaling (83, 84, 136). These intense energy requirements make axons, particularly in unmyelinated regions, highly vulnerable to dysfunction in mitochondria, the organelles responsible for generating ATP to power activities within a cell (45). Mitochondrial dysfunction is thought to contribute to major neurodegenerative diseases including Alzheimer’s disease, Parkinson’s disease, Huntington’s disease, and amyotrophic lateral sclerosis (137–140). The optic nerve in particular is especially vulnerable to the metabolic consequences of mitochondrial dysfunction, making it the primary site of neurological damage in mitochondrial disorders such as Leber’s hereditary optic neuropathy and dominant optic atrophy, known collectively as Mitochondrial Optic Neuropathies (MON) (83, 84, 141). Due to its length and energy demands both along the axon and at the synapse, mitochondria are located throughout the optic nerve in regions of high energy utilization, and unmyelinated axons at the optic nerve head exhibit particularly high mitochondrial density (14, 83, 142–144). However, mitochondria are impermanent structures that must be routinely replaced, and deficits in mitophagy – the process of eliminating aged or damaged mitochondria – can result in decreased energy utilization and the exposure of the cell to damaging ROS and other factors (83, 145).

Historically cells were thought to degrade their own mitochondria, but in the last decade a population of phagocytic astrocytes at the optic nerve head has been shown to aid in the degradation of axonal mitochondria (55, 56). These astrocytes constitutively express the phagocytic marker galectin-3 (Lgals3/Mac-2) and engage in a process termed transmitophagy, in which mitochondria are extruded by axons before being engulfed and eliminated by astrocytes. This elimination of axonal mitochondria by phagocytic ONH astrocytes is estimated to exceed mitophagy by RGCs in the ganglion cell layer, despite greater abundance of mitochondria in that region, suggesting that distal axonal mitochondria may undergo retrograde transport to this region specifically for degradation. Consistent with previous studies showing galectin-3 expression by reactive astrocytes in mouse models of ischemia (75, 146) and physical injury (147) – as well as from patients with the demyelinating disease multiple sclerosis (148) – expression at the ONH rises in both DBA/2J and inducible OHT mouse models of glaucoma (55), and has been detected at the ONH in human glaucomatous tissue (149).

Curiously, work by other groups in both DBA/2J and microbead-induced mouse models of glaucoma shows an increased percentage of axonal mitochondria with indicators of damage, such as depleted cristae, in this region (150, 151). The increase in a marker of phagocytic capacity (galectin-3) combined with accumulation of degradation-bound mitochondria, although superficially paradoxical, may aid in identifying the contributions of phagocytic and mitochondrial dysfunction in glaucoma. Defects in axonal transport, both anterograde and retrograde, are well established in glaucoma and emerge early in inducible OHT models (14, 152), and a recently published study identifies a decrease in anterograde transport of mitochondria and an accompanying decline in axonal ATP in a mouse microbead model (153). One possibility is that axonal transport disruption impairs the replacement of damaged mitochondria, leading to their accumulation and worsening metabolic dysfunction. An alternative is suggested by early work on non-human primates implicating ONH astrocytes in axonal phagocytosis in a model of glaucoma (154, 155). Astrocytes may have a more limited phagocytic capacity than professional phagocytes such as microglia and macrophages, and the need to eliminate axonal debris as glaucoma progresses may exceed this capacity and result in a ‘backlog’ of damaged mitochondria (156).

Regardless of the underlying cause, an accumulation of damaged mitochondria in the ONH would be expected to exacerbate energy deficits in the region and lead to the increased production of reactive oxygen species and oxidative stress (120), contributing to the indicators of neuroinflammation seen in glaucoma (12). Furthermore, the group responsible for identifying transmitophagy in the optic nerve also claims to have identified potential sites of this function in the cortex, and recent work in a mouse model of Parkinson’s Disease suggests this may indeed be a specialized astrocyte function elsewhere in the CNS (56, 157). Given the evidence for a link between mitochondrial dysfunction and neurodegenerative disorders, it may be that disruption of transmitophagy plays a role in neurodegeneration more broadly. Finally, upregulation of galectin-3 is also found in an activated microglia state associated with neurodegeneration (158, 159) and may act to propagate the activated state to nearby microglia (160); moreover, recent work has shown that disrupting its expression is protective in a mouse model of glaucoma (161). Thus, we should consider the possibility that astrocytic upregulation of galectin-3 potentiates the microglial (162) and/or peripheral immune (163, 164) response in glaucoma, which may contribute to a neuroinflammation in the region.

A number of related questions regarding mitochondrial function and turnover in glaucoma remain unanswered. Astrocytes are known to utilize at least 2 distinct phagocytic pathways - MEGF10 and MERTK – but which, if either, is active at the ONH remains unknown; identifying the phagocytic machinery expressed by this astrocyte population may improve our understanding of the transmitophagic process under physiological and pathological conditions (146, 165). While our transcriptional data confirms high expression of Lgals3 in astrocytes of this region, it unfortunately does not clarify what other factors may be involved (90). Furthermore, just as neurons have been shown to utilize astrocytes for elimination of degraded mitochondria, evidence in a mouse model of stroke has shown that astrocytes donate healthy mitochondria to neurons after injury (166). It remains unknown whether a similar behavior occurs in the optic nerve; although our data shows an increased expression of mitochondrial components by astrocytes in this region that would be consistent with such a phenomenon, it is unable to conclusively demonstrate this possibility. Finally, at least one approach for neuroprotection in glaucoma involves the supplementation of nicotinamide (vitamin B3), a precursor of the metabolic cofactor NAD^+^, which is vital for mitochondrial function but declines with age (167). Oral supplementation in the DBA/2J mouse model of glaucoma protected RGCs from glaucomatous progression, and human trials are ongoing to assess the viability of this approach at a clinical level (168).

## Long range energy provisioning via the optic nerve astrocyte network

In times of inadequate provisioning - due to hypoglycemia or elevated metabolic demand - astrocytic glycogen stores provide a repository of usable energy within the nervous system that can preserve neuronal function until equilibrium is restored between metabolic needs and substrate availability (23). The sequestration of glycogen by astrocytes, although low by standards of other body tissues, is extensively documented, though much regarding the mechanisms of provisioning remains unresolved (169, 170). A leading hypothesis, the astrocyte-neuron lactate shuttle, states that astrocytic glycogen is converted to lactate and secreted to neurons through monocarboxylate transporters, but aspects of the hypothesis remain contentious (20, 171, 172). Previous studies suggest key differences in gray and white matter astrocyte provisioning, including the metabolic signals that induces substrate transfer (glutamate in the former, while K^+^ is a primary candidate in the later) and indicate that white matter astrocytes may store lower levels of glycogen than those of gray matter (37, 171, 173, 174).

The optic nerve has several properties that make it a favored model for studying energy provisioning from astrocytes to axons in white matter: surgical accessibility, the presence of axons and glia but no neuronal soma, and a straightforward anatomical structure (23, 170). It is also a key site of disease progression in glaucoma, as well as a number of mitochondrial optic neuropathies, a group of genetic disorders that alter mitochondrial function throughout the body but primarily damage retinal ganglion cell axons, suggesting it is amongst the most metabolically vulnerable of tissues (14, 83, 84, 141, 142, 175). As such, the optic nerve is a frequently utilized system for *ex vivo* investigation of astrocytic and axonal metabolomics (129, 176). Prior work in the optic nerve has established an essential role for astrocytic glycogen in maintaining axonal function during conditions of glucose depletion or elevated signaling activity, with experimental manipulation of glycogen levels showing that pre-depletion lessens the duration of preserved function; similar results have been demonstrated with gray matter astrocytes of the hippocampus in *ex vivo* slices (129, 177). However, until recently direct evidence for white matter astrocytic-glycogen support *in vivo* has been limited and investigations have typically involved short periods of intense glucose deprivation, rather than extended periods of modest deprivation that may be more representative of disease states.

A recently published study of energy provisioning from astrocytic glycogen stores in the optic nerve shows the relevance of this phenomenon *in vivo* in a microbead occlusion mouse model of glaucoma (57). Unlike previous *ex vivo* studies of optic nerve glycogen utilization, the moderate (~35%) IOP elevation provides a stimulus more representative of glaucomatous insult, and one in which glycogen preserves axonal function over the course of days and weeks, rather than minutes and hours (129, 170). This study is particularly notable for several reasons:

1. Indication of an early energy deficit in OHT optic nerve – detectable by day 4 as a decline in glycogen reserves, and an increase in phosphorylation of the cellular energy sensor AMPK,
2. *in vivo* demonstration of a role for astrocytic glycogen in preserving optic nerve function, with functional losses at the behavioral level and in axonal transport occurring more rapidly in glycogen depleted nerves, and
3. A clear role for the gap junction coupled astrocyte network in energy provisioning across anatomically significant distances (≥ 10mm).

The most striking of these results is the long distance, Cx43-mediated provisioning of energy substrates, in which glycogen reserves in the contralateral nerve appear to metabolically buffer the optic nerve of the microbead-treated eye, helping to preserve axonal function at the expense of depleting reserves and increasing metabolic vulnerability in the donating nerve. When mice with astrocyte-specific Cx43 deletion were treated unilaterally with microbeads, they experienced more rapid declines in function and glycogen levels in the ipsilateral nerve, while the contralateral nerve retained greater glycogen reserves and remained less sensitive to future insults. These mice also demonstrated more rapid loss of axonal transport function than WT animals, which show minimal decline in either nerve one week after microbead injection, whereas KO animals show an approximately 40% reduction in the ipsilateral nerve. As with prior *ex vivo* studies, the greater the stores of glycogen available, the longer axons were able to continue functioning with only modest deficits, demonstrating a neuroprotective effect for astrocytic glycogen provisioning *in vivo*.

The implication of the Cx43 coupled astrocyte network in these results also suggests a potential mechanism for the previously observed phenomenon of astrocyte reactivity spreading from injured eye to contralateral retina (110). Cx43 is upregulated in human glaucoma, both in retinal astrocytes and at the ONH, and multiple groups have claimed that gap junctions may propagate deleterious signals in neurodegeneration and injury (112, 178, 179). However, the outcome of this study – Cx43-mediated preservation of function in injured tissue at the expense of increased vulnerability in distal regions – suggests a more nuanced interpretation is in order.

While axonal dysfunction – including disrupted axonal transport and poor mitochondrial health – are thought to be early components of glaucoma, these results suggest that the depletion of astrocytic energy stores in the optic nerve also plays an early role (9, 83, 84, 152). In keeping with this interpretation, work published in 2018 found significant depletion of glycogen in aged DBA/2J mouse optic nerves relative to age matched controls, and DBA/2J and microbead-treated mouse models showed an increase in phosphorylation of AMPK – a marker of metabolic stress – in optic nerve astrocytes; similar changes in AMPK phosphorylation were also observed in tissue from glaucomatous human optic nerve (180). These results suggest an energy deficit, congruent with reports of metabolic dysfunction in glaucoma, resulting from decreased energy utilization and/or elevated metabolic costs.

Intriguingly, the 2018 study showed a decrease in lactate transferring monocarboxylate transporters in optic nerve astrocytes and RGC axons in both DBA/2J mice and mouse microbead models, as well as a decrease in axonal monocarboxylate transporters in human glaucoma (180). These counterintuitive findings – a decline in components of astrocyte to neuron energy provisioning during a period of elevated axonal vulnerability – may point the way to avenues of research that shed light on the function of astrocytes in glaucoma and beyond. Some work suggests that reactivity may modulate ‘self-preservation’ pathways in astrocytes, potentially diminishing their ability to aid distressed neurons (181–184). Although seemingly disadvantageous from the viewpoint of neuroprotection, acute focal injuries in the CNS – wherein a penumbral zone populated by glia but depleted of neurons surrounds the infarct core and separates it from healthy neural parenchyma – provide examples of scenarios in which such an adaptation would be a net advantage for organismal survival (185, 186). Alternatively, damage to mitochondria may render them unable to effectively utilize lactate from astrocytes, cutting off axons from this energy source and exacerbating metabolic dysfunction. Given that energy dysfunction and propagation of neurodegeneration are implicated in a variety of neurological injuries and diseases, developing a greater understanding of the networked behavior of astrocytes may provide future avenues for early intervention in these devastating conditions.

## Conclusion

Although the heterogeneity of astrocyte functions as a research avenue is still at a formative stage – particularly with regard to white matter, fibrous astrocytes – it is a topic that is likely to play an outsized role as the field moves from foundational research to the consideration of astrocytes as druggable targets in specific neurological injuries and neurodegenerative diseases such as glaucoma. Given the unique vulnerability of retinal ganglion cell axons, especially to metabolic disruption, and the specialized role localized populations of astrocytes play in supporting these axons, we expect a more complete understanding of neuron-glia coordination in the anterior visual pathway to reveal future avenues for neuroprotective treatment that can supplement or replace the current approach of IOP management. Finally, although we advise caution in inferring behaviors across astrocyte populations, the accessibility of the anterior visual pathway may make it an ideal region for future investigations of white matter astrocytes and the development of novel approaches to elucidate their behavior.

## References

[1] Tham YC, Li X, Wong TY, Quigley HA, Aung T, Cheng CY. Global prevalence of glaucoma and projections of glaucoma burden through 2040: a systematic review and meta-analysis. Ophthalmology (2014) 121:2081–90. doi: 10.1016/j.ophtha.2014.05.013 24974815

[2] Weinreb RN, Leung CK, Crowston JG, Medeiros FA, Friedman DS, Wiggs JL, Primary open-angle glaucoma. Nat Rev Dis Primers (2016) 2:16067. doi: 10.1038/nrdp.2016.67 27654570

[3] Alqawlaq S, Flanagan JG, Sivak JM. All roads lead to glaucoma: Induced retinal injury cascades contribute to a common neurodegenerative outcome. Exp Eye Res (2019) 183:88–97. doi: 10.1016/j.exer.2018.11.005 30447198

[4] Rein DB, Zhang P, Wirth KE, Lee PP, Hoerger TJ, McCall N, The economic burden of major adult visual disorders in the United States. Arch Ophthalmol (2006) 124:1754–60. doi: 10.1001/archopht.124.12.1754 17159036

[5] Varma R, Lee PP, Goldberg I, Kotak S. An assessment of the health and economic burdens of glaucoma. Am J Ophthalmol (2011) 152:515–22. doi: 10.1016/j.ajo.2011.06.004 21961848 PMC3206636

[6] Weinreb RN, Aung T, Medeiros FA. The pathophysiology and treatment of glaucoma: a review. JAMA (2014) 311:1901–11. doi: 10.1001/jama.2014.3192 24825645 PMC4523637

[7] Baudouin C, Kolko M, Melik-Parsadaniantz S, Messmer EM. Inflammation in Glaucoma: From the back to the front of the eye, and beyond. Prog Retin Eye Res (2021) 83:100916. doi: 10.1016/j.preteyeres.2020.100916 33075485

[8] Heijl A, Buchholz P, Norrgren G, Bengtsson B. Rates of visual field progression in clinical glaucoma care. Acta Ophthalmol (2013) 91:406–12. doi: 10.1111/j.1755-3768.2012.02492.x 23066646 PMC3798127

[9] Quigley HA. Understanding glaucomatous optic neuropathy: the synergy between clinical observation and investigation. Annu Rev Vis Sci (2016) 2:235–54. doi: 10.1146/annurev-vision-111815-114417 28532352

[10] Hernandez MR, Pena JD. The optic nerve head in glaucomatous optic neuropathy. Arch Ophthalmol (1997) 115:389–95. doi: 10.1001/archopht.1997.01100150391013 9076213

[11] Schneider M, Fuchshofer R. The role of astrocytes in optic nerve head fibrosis in glaucoma. Exp Eye Res (2016) 142:49–55. doi: 10.1016/j.exer.2015.08.014 26321510

[12] Tezel G. Molecular regulation of neuroinflammation in glaucoma: Current knowledge and the ongoing search for new treatment targets. Prog Retin Eye Res (2022) 87:100998. doi: 10.1016/j.preteyeres.2021.100998 34348167 PMC8803988

[13] Hernandez MR. The optic nerve head in glaucoma: role of astrocytes in tissue remodeling. Prog Retinal Eye Res (2000) 19:297–321. doi: 10.1016/S1350-9462(99)00017-8 10749379

[14] Yu DY, Cringle SJ, Balaratnasingam C, Morgan WH, Yu PK, Su EN. Retinal ganglion cells: Energetics, compartmentation, axonal transport, cytoskeletons and vulnerability. Prog Retin Eye Res (2013) 36:217–46. doi: 10.1016/j.preteyeres.2013.07.001 23891817

[15] Jakel S, Dimou L. Glial cells and their function in the adult brain: A journey through the history of their ablation. Front Cell Neurosci (2017) 11:24. doi: 10.3389/fncel.2017.00024 28243193 PMC5303749

[16] Sofroniew MV, Vinters HV. Astrocytes: biology and pathology. Acta Neuropathol (2010) 119:7–35. doi: 10.1007/s00401-009-0619-8 20012068 PMC2799634

[17] Verkhratsky A, Parpura V, Vardjan N, Zorec R. Physiology of astroglia. Adv Exp Med Biol (2019) 1175:45–91. doi: 10.1007/978-981-13-9913-8_3 31583584 PMC7265998

[18] Barres BA. The mystery and magic of glia: a perspective on their roles in health and disease. Neuron (2008) 60:430–40. doi: 10.1016/j.neuron.2008.10.013 18995817

[19] Abbott NJ, Ronnback L, Hansson E. Astrocyte-endothelial interactions at the blood-brain barrier. Nat Rev Neurosci (2006) 7:41–53. doi: 10.1038/nrn1824 16371949

[20] Magistretti PJ, Allaman I. A cellular perspective on brain energy metabolism and functional imaging. Neuron (2015) 86:883–901. doi: 10.1016/j.neuron.2015.03.035 25996133

[21] Kofuji P, Newman EA. Potassium buffering in the central nervous system. Neuroscience (2004) 129:1045–56. doi: 10.1016/j.neuroscience.2004.06.008 15561419 PMC2322935

[22] Mahmoud S, Gharagozloo M, Simard C, Gris D. Astrocytes maintain glutamate homeostasis in the CNS by controlling the balance between glutamate uptake and release. Cells (2019) 8:184. doi: 10.3390/cells8020184 30791579 PMC6406900

[23] Brown AM, Ransom BR. Astrocyte glycogen and brain energy metabolism. Glia (2007) 55:1263–71. doi: 10.1002/glia.20557 17659525

[24] Bellot-Saez A, Kekesi O, Morley JW, Buskila Y. Astrocytic modulation of neuronal excitability through K(+) spatial buffering. Neurosci Biobehav Rev (2017) 77:87–97. doi: 10.1016/j.neubiorev.2017.03.002 28279812

[25] Giaume C, Koulakoff A, Roux L, Holcman D, Rouach N. Astroglial networks: a step further in neuroglial and gliovascular interactions. Nat Rev Neurosci (2010) 11:87–99. doi: 10.1038/nrn2757 20087359

[26] Jha MK, Kim JH, Song GJ, Lee WH, Lee IK, Lee HW, Functional dissection of astrocyte-secreted proteins: Implications in brain health and diseases. Prog Neurobiol (2018) 162:37–69. doi: 10.1016/j.pneurobio.2017.12.003 29247683

[27] Poyhonen S, Er S, Domanskyi A, Airavaara M. Effects of neurotrophic factors in glial cells in the central nervous system: expression and properties in neurodegeneration and injury. Front Physiol (2019) 10:486. doi: 10.3389/fphys.2019.00486 31105589 PMC6499070

[28] Allen NJ, Eroglu C. Cell biology of astrocyte-synapse interactions. Neuron (2017) 96:697–708. doi: 10.1016/j.neuron.2017.09.056 29096081 PMC5687890

[29] Livne-Bar I, Wei J, Liu HH, Alqawlaq S, Won GJ, Tuccitto A, Astrocyte-derived lipoxins A4 and B4 promote neuroprotection from acute and chronic injury. J Clin Invest (2017) 127:4403–14. doi: 10.1172/JCI77398 29106385 PMC5707141

[30] Guttenplan KA, Weigel MK, Prakash P, Wijewardhane PR, Hasel P, Rufen-Blanchette U, Neurotoxic reactive astrocytes induce cell death via saturated lipids. Nature (2021) 599:102–7. doi: 10.1038/s41586-021-03960-y 34616039 PMC12054010

[31] Flavia T, Maria A, Cristina L. Chemokines: key molecules that orchestrate communication among neurons, microglia and astrocytes to preserve brain function. Neuroscience (2020) 439:230–40. doi: 10.1016/j.neuroscience.2019.07.035 31376422

[32] Zhang Y, Barres BA. Astrocyte heterogeneity: an underappreciated topic in neurobiology. Curr Opin Neurobiol (2010) 20:588–94. doi: 10.1016/j.conb.2010.06.005 20655735

[33] Khakh BS, Deneen B. The emerging nature of astrocyte diversity. Annu Rev Neurosci (2019) 42:187–207. doi: 10.1146/annurev-neuro-070918-050443 31283899

[34] Pestana F, Edwards-Faret G, Belgard TG, Martirosyan A, Holt MG. No longer underappreciated: the emerging concept of astrocyte heterogeneity in neuroscience. Brain Sci (2020) 10:168. doi: 10.3390/brainsci10030168 32183137 PMC7139801

[35] Ben Haim L, Rowitch DH. Functional diversity of astrocytes in neural circuit regulation. Nat Rev Neurosci (2017) 18:31–41. doi: 10.1038/nrn.2016.159 27904142

[36] Andriezen WL. The neuroglia elements in the human brain. Br Med J (1893) 2:227–30. doi: 10.1136/bmj.2.1700.227 PMC2422013 20754383

[37] Kohler S, Winkler U, Hirrlinger J. Heterogeneity of astrocytes in grey and white matter. Neurochem Res (2021) 46:3–14. doi: 10.1007/s11064-019-02926-x 31797158

[38] Chung WS, Allen NJ, Eroglu C. Astrocytes control synapse formation, function, and elimination. Cold Spring Harb Perspect Biol (2015) 7:a020370. doi: 10.1101/cshperspect.a020370 25663667 PMC4527946

[39] Perea G, Navarrete M, Araque A. Tripartite synapses: astrocytes process and control synaptic information. Trends Neurosci (2009) 32:421–31. doi: 10.1016/j.tins.2009.05.001 19615761

[40] Black JA, Waxman SG. The perinodal astrocyte. Glia (1988) 1:169–83. doi: 10.1002/glia.440010302 2976037

[41] Butt AM, Pugh M, Hubbard P, James G. Functions of optic nerve glia: axoglial signalling in physiology and pathology. Eye (Lond) (2004) 18:1110–21. doi: 10.1038/sj.eye.6701595 15534596

[42] Dutta DJ, Woo DH, Lee PR, Pajevic S, Bukalo O, Huffman WC, Regulation of myelin structure and conduction velocity by perinodal astrocytes. Proc Natl Acad Sci U.S.A (2018) 115:11832–7. doi: 10.1073/pnas.1811013115 30373833 PMC6243273

[43] Orthmann-Murphy JL, Abrams CK, Scherer SS. Gap junctions couple astrocytes and oligodendrocytes. J Mol Neurosci (2008) 35:101–16. doi: 10.1007/s12031-007-9027-5 18236012 PMC2650399

[44] Lundgaard I, Osorio MJ, Kress BT, Sanggaard S, Nedergaard M. White matter astrocytes in health and disease. Neuroscience (2014) 276:161–73. doi: 10.1016/j.neuroscience.2013.10.050 24231735 PMC4016995

[45] Ito YA, Di Polo A. Mitochondrial dynamics, transport, and quality control: A bottleneck for retinal ganglion cell viability in optic neuropathies. Mitochondrion (2017) 36:186–92. doi: 10.1016/j.mito.2017.08.014 28866056

[46] Wender R, Brown AM, Fern R, Swanson RA, Farrell K, Ransom BR. Astrocytic glycogen influences axon function and survival during glucose deprivation in central white matter. J Neurosci (2000) 20:6804–10. doi: 10.1523/JNEUROSCI.20-18-06804.2000 10995824 PMC6772835

[47] Mishra A, Reynolds JP, Chen Y, Gourine AV, Rusakov DA, Attwell D. Astrocytes mediate neurovascular signaling to capillary pericytes but not to arterioles. Nat Neurosci (2016) 19:1619–27. doi: 10.1038/nn.4428 27775719 PMC5131849

[48] Gourine AV, Kasymov V, Marina N, Tang F, Figueiredo MF, Lane S, Astrocytes control breathing through pH-dependent release of ATP. Science (2010) 329:571–5. doi: 10.1126/science.1190721 20647426 PMC3160742

[49] Brancaccio M, Edwards MD, Patton AP, Smyllie NJ, Chesham JE, Maywood ES, Cell-autonomous clock of astrocytes drives circadian behavior in mammals. Science (2019) 363:187–92. doi: 10.1126/science.aat4104 30630934 PMC6440650

[50] Lanjakornsiripan D, Pior BJ, Kawaguchi D, Furutachi S, Tahara T, Katsuyama Y, Layer-specific morphological and molecular differences in neocortical astrocytes and their dependence on neuronal layers. Nat Commun (2018) 9:1623. doi: 10.1038/s41467-018-03940-3 29691400 PMC5915416

[51] Batiuk MY, Martirosyan A, Wahis J, de Vin F, Marneffe C, Kusserow C, Identification of region-specific astrocyte subtypes at single cell resolution. Nat Commun (2020) 11:1220. doi: 10.1038/s41467-019-14198-8 32139688 PMC7058027

[52] Ye H, Hernandez MR. Heterogeneity of astrocytes in human optic nerve head. J Comp Neurol (1995) 362:441–52. doi: 10.1002/cne.903620402 8636460

[53] Sun D, Lye-Barthel M, Masland RH, Jakobs TC. The morphology and spatial arrangement of astrocytes in the optic nerve head of the mouse. J Comp Neurol (2009) 516:1–19. doi: 10.1002/cne.22058 19562764 PMC2900161

[54] Nagelhus EA, Veruki ML, Torp R, Haug F-M, Laake JH, Nielsen S, Aquaporin-4 water channel protein in the rat retina and optic nerve: polarized expression in müller cells and fibrous astrocytes. J Neurosci (1998) 18:2506–19. doi: 10.1523/JNEUROSCI.18-07-02506.1998 9502811 PMC6793100

[55] Nguyen JV, Soto I, Kim KY, Bushong EA, Oglesby E, Valiente-Soriano FJ, Myelination transition zone astrocytes are constitutively phagocytic and have synuclein dependent reactivity in glaucoma. Proc Natl Acad Sci U.S.A (2011) 108:1176–81. doi: 10.1073/pnas.1013965108 21199938 PMC3024691

[56] Davis CH, Kim KY, Bushong EA, Mills EA, Boassa D, Shih T, Transcellular degradation of axonal mitochondria. Proc Natl Acad Sci U.S.A (2014) 111:9633–8. doi: 10.1073/pnas.1404651111 24979790 PMC4084443

[57] Cooper ML, Pasini S, Lambert WS, D’Alessandro KB, Yao V, Risner ML, Redistribution of metabolic resources through astrocyte networks mitigates neurodegenerative stress. Proc Natl Acad Sci U.S.A (2020) 117:18810–21. doi: 10.1073/pnas.2009425117 32690710 PMC7414143

[58] Pekny M, Pekna M. Astrocyte reactivity and reactive astrogliosis: costs and benefits. Physiol Rev (2014) 94:1077–98. doi: 10.1152/physrev.00041.2013 25287860

[59] Sofroniew MV. Astrocyte reactivity: subtypes, states, and functions in CNS innate immunity. Trends Immunol (2020) 41:758–70. doi: 10.1016/j.it.2020.07.004 32819810 PMC7484257

[60] Moulson AJ, Squair JW, Franklin RJM, Tetzlaff W, Assinck P. Diversity of reactive astrogliosis in CNS pathology: heterogeneity or plasticity? Front Cell Neurosci (2021) 15:703810. doi: 10.3389/fncel.2021.703810 34381334 PMC8349991

[61] Ben Haim L, Carrillo-de Sauvage MA, Ceyzeriat K, Escartin C. Elusive roles for reactive astrocytes in neurodegenerative diseases. Front Cell Neurosci (2015) 9:278. doi: 10.3389/fncel.2015.00278 26283915 PMC4522610

[62] Garcia-Bermudez MY, Freude KK, Mouhammad ZA, van Wijngaarden P, Martin KK, Kolko M. Glial cells in glaucoma: friends, foes, and potential therapeutic targets. Front Neurol (2021) 12:624983. doi: 10.3389/fneur.2021.624983 33796062 PMC8007906

[63] Boal AM, Risner ML, Cooper ML, Wareham LK, Calkins DJ. Astrocyte networks as therapeutic targets in glaucomatous neurodegeneration. Cells (2021) 10:1368. doi: 10.3390/cells10061368 34199470 PMC8228804

[64] de Hoz R, Rojas B, Ramirez AI, Salazar JJ, Gallego BI, Trivino A, Retinal macroglial responses in health and disease. BioMed Res Int (2016) 2016:2954721. doi: 10.1155/2016/2954721 27294114 PMC4887628

[65] Heneka MT, Carson MJ, El Khoury J, Landreth GE, Brosseron F, Feinstein DL, Neuroinflammation in alzheimer’s disease. Lancet Neurol (2015) 14:388–405. doi: 10.1016/S1474-4422(15)70016-5 25792098 PMC5909703

[66] Simon DW, McGeachy MJ, Bayir H, Clark RS, Loane DJ, Kochanek PM. The far-reaching scope of neuroinflammation after traumatic brain injury. Nat Rev Neurol (2017) 13:171–91. doi: 10.1038/nrneurol.2017.13 28186177 PMC5675525

[67] DiSabato DJ, Quan N, Godbout JP. Neuroinflammation: the devil is in the details. J Neurochem (2016) 139 Suppl 2:136–53. doi: 10.1111/jnc.13607 26990767 PMC5025335

[68] Frank-Cannon TC, Alto LT, McAlpine FE, Tansey MG. Does neuroinflammation fan the flame in neurodegenerative diseases? Mol Neurodegener (2009) 4:47. doi: 10.1186/1750-1326-4-47 19917131 PMC2784760

[69] Escartin C, Galea E, Lakatos A, O’Callaghan JP, Petzold GC, Serrano-Pozo A, Reactive astrocyte nomenclature, definitions, and future directions. Nat Neurosci (2021) 24:312–325. doi: 10.1038/s41593-020-00783-4 33589835 PMC8007081

[70] Silver J, Miller JH. Regeneration beyond the glial scar. Nat Rev Neurosci (2004) 5:146–56. doi: 10.1038/nrn1326 14735117

[71] Sofroniew MV. Molecular dissection of reactive astrogliosis and glial scar formation. Trends Neurosci (2009) 32:638–47. doi: 10.1016/j.tins.2009.08.002 19782411 PMC2787735

[72] Anderson MA, Burda JE, Ren Y, Ao Y, O’Shea TM, Kawaguchi R, Astrocyte scar formation aids central nervous system axon regeneration. Nature (2016) 532:195–200. doi: 10.1038/nature17623 27027288 PMC5243141

[73] Voskuhl RR, Peterson RS, Song B, Ao Y, Morales LB, Tiwari-Woodruff S, Reactive astrocytes form scar-like perivascular barriers to leukocytes during adaptive immune inflammation of the CNS. J Neurosci (2009) 29:11511–22. doi: 10.1523/JNEUROSCI.1514-09.2009 19759299 PMC2768309

[74] Sun D, Moore S, Jakobs TC. Optic nerve astrocyte reactivity protects function in experimental glaucoma and other nerve injuries. J Exp Med (2017) 214:1411–30. doi: 10.1084/jem.20160412 28416649 PMC5413323

[75] Zamanian JL, Xu L, Foo LC, Nouri N, Zhou L, Giffard RG, Genomic analysis of reactive astrogliosis. J Neurosci (2012) 32:6391–410. doi: 10.1523/JNEUROSCI.6221-11.2012 22553043 PMC3480225

[76] Liddelow SA, Guttenplan KA, Clarke LE, Bennett FC, Bohlen CJ, Schirmer L, Neurotoxic reactive astrocytes are induced by activated microglia. Nature (2017) 541:481–7. doi: 10.1038/nature21029 28099414 PMC5404890

[77] Al-Dalahmah O, Sosunov AA, Shaik A, Ofori K, Liu Y, Vonsattel JP, Single-nucleus RNA-seq identifies Huntington disease astrocyte states. Acta Neuropathol Commun (2020) 8:19. doi: 10.1186/s40478-020-0880-6 32070434 PMC7029580

[78] Grubman A, Chew G, Ouyang JF, Sun G, Choo XY, McLean C, A single-cell atlas of entorhinal cortex from individuals with Alzheimer’s disease reveals cell-type-specific gene expression regulation. Nat Neurosci (2019) 22:2087–97. doi: 10.1038/s41593-019-0539-4 31768052

[79] Das S, Li Z, Noori A, Hyman BT, Serrano-Pozo A. Meta-analysis of mouse transcriptomic studies supports a context-dependent astrocyte reaction in acute CNS injury versus neurodegeneration. J Neuroinflamm (2020) 17:227. doi: 10.1186/s12974-020-01898-y PMC7393869 32736565

[80] Clarke LE, Liddelow SA, Chakraborty C, Munch AE, Heiman M, Barres BA. Normal aging induces A1-like astrocyte reactivity. Proc Natl Acad Sci U.S.A (2018) 115:E1896–905. doi: 10.1073/pnas.1800165115 29437957 PMC5828643

[81] Boisvert MM, Erikson GA, Shokhirev MN, Allen NJ. The aging astrocyte transcriptome from multiple regions of the mouse brain. Cell Rep (2018) 22:269–85. doi: 10.1016/j.celrep.2017.12.039 29298427 PMC5783200

[82] Matias I, Morgado J, Gomes FCA. Astrocyte heterogeneity: impact to brain aging and disease. Front Aging Neurosci (2019) 11:59. doi: 10.3389/fnagi.2019.00059 30941031 PMC6433753

[83] Muench NA, Patel S, Maes ME, Donahue RJ, Ikeda A, Nickells RW. The influence of mitochondrial dynamics and function on retinal ganglion cell susceptibility in optic nerve disease. Cells (2021) 10:1593. doi: 10.3390/cells10071593 34201955 PMC8306483

[84] Inman DM, Harun-Or-Rashid M. Metabolic vulnerability in the neurodegenerative disease glaucoma. Front Neurosci (2017) 11:146. doi: 10.3389/fnins.2017.00146 28424571 PMC5371671

[85] Wang R, Seifert P, Jakobs TC. Astrocytes in the optic nerve head of glaucomatous mice display a characteristic reactive phenotype. Invest Ophthalmol Vis Sci (2017) 58:924–32. doi: 10.1167/iovs.16-20571 28170536 PMC5300248

[86] Skoff RP. Gliogenesis in rat optic-nerve - astrocytes are generated in a single wave before oligodendrocytes. Dev Biol (1990) 139:149–68. doi: 10.1016/0012-1606(90)90285-Q 2328833

[87] Watanabe T, Raff MC. Retinal astrocytes are immigrants from the optic nerve. Nature (1988) 332:834–7. doi: 10.1038/332834a0 3282180

[88] Ling TL, Mitrofanis J, Stone J. Origin of retinal astrocytes in the rat: evidence of migration from the optic nerve. J Comp Neurol (1989) 286:345–52. doi: 10.1002/cne.902860305 2768562

[89] Kimball EC, Quillen S, Pease ME, Keuthan C, Nagalingam A, Zack DJ, Aquaporin 4 is not present in normal porcine and human lamina cribrosa. PloS One (2022) 17:e0268541. doi: 10.1371/journal.pone.0268541 35709078 PMC9202842

[90] Mazumder AG, Jule AM, Cullen PF, Sun D. Astrocyte heterogeneity within white matter tracts and a unique subpopulation of optic nerve head astrocytes. iScience (2022) 25:105568. doi: 10.1016/j.isci.2022.105568 36465110 PMC9708923

[91] Selvam S, Kumar T, Fruttiger M. Retinal vasculature development in health and disease. Prog Retin Eye Res (2018) 63:1–19. doi: 10.1016/j.preteyeres.2017.11.001 29129724

[92] Fruttiger M, Calver AR, Kruger WH, Mudhar HS, Michalovich D, Takakura N, PDGF mediates a neuron-astrocyte interaction in the developing retina. Neuron (1996) 17:1117–31. doi: 10.1016/S0896-6273(00)80244-5 8982160

[93] Stone J, Dreher Z. Relationship between astrocytes, ganglion-cells and vasculature of the retina. J Comp Neurol (1987) 255:35–49. doi: 10.1002/cne.902550104 3819008

[94] Tao C, Zhang X. Development of astrocytes in the vertebrate eye. Dev Dyn (2014) 243:1501–10. doi: 10.1002/dvdy.24190 25236977 PMC4237691

[95] Biesecker KR, Srienc AI, Shimoda AM, Agarwal A, Bergles DE, Kofuji P, Glial cell calcium signaling mediates capillary regulation of blood flow in the retina. J Neurosci (2016) 36:9435–45. doi: 10.1523/JNEUROSCI.1782-16.2016 27605617 PMC5013190

[96] Hollander H, Makarov F, Dreher Z, van Driel D, Chan-Ling TL, Stone J. Structure of the macroglia of the retina: sharing and division of labour between astrocytes and Muller cells. J Comp Neurol (1991) 313:587–603. doi: 10.1002/cne.903130405 1783683

[97] Ogden TE. Nerve fiber layer astrocytes of the primate retina: morphology, distribution, and density. Invest Ophthalmol Vis Sci (1978) 17:499–510. 96038

[98] Ramirez JM, Trivino A, Ramirez AI, Salazar JJ, Garcia-Sanchez J. Immunohistochemical study of human retinal astroglia. Vision Res (1994) 34:1935–46. doi: 10.1016/0042-6989(94)90024-8 7941395

[99] Yan W, Peng YR, van Zyl T, Regev A, Shekhar K, Juric D, Cell atlas of the human fovea and peripheral retina. Sci Rep (2020) 10:9802. doi: 10.1038/s41598-020-66092-9 32555229 PMC7299956

[100] Lukowski SW, Lo CY, Sharov AA, Nguyen Q, Fang L, Hung SS, A single-cell transcriptome atlas of the adult human retina. EMBO J (2019) 38:e100811. doi: 10.15252/embj.2018100811 31436334 PMC6745503

[101] Menon M, Mohammadi S, Davila-Velderrain J, Goods BA, Cadwell TD, Xing Y, Single-cell transcriptomic atlas of the human retina identifies cell types associated with age-related macular degeneration. Nat Commun (2019) 10:4902. doi: 10.1038/s41467-019-12780-8 31653841 PMC6814749

[102] Zahs KR, Wu T. Confocal microscopic study of glial-vascular relationships in the retinas of pigmented rats. J Comp Neurol (2001) 429:253–69. doi: 10.1002/1096-9861(20000108)429:2<253::AID-CNE6>3.0.CO;2-S 11116218

[103] Jammalamadaka A, Suwannatat P, Fisher SK, Manjunath BS, Hollerer T, Luna G. Characterizing spatial distributions of astrocytes in the mammalian retina. Bioinformatics (2015) 31:2024–31. doi: 10.1093/bioinformatics/btv097 25686636

[104] Punal VM, Paisley CE, Brecha FS, Lee MA, Perelli RM, Wang J, Large-scale death of retinal astrocytes during normal development is non-apoptotic and implemented by microglia. PloS Biol (2019) 17:e3000492. doi: 10.1371/journal.pbio.3000492 31626642 PMC6821132

[105] Wang J, O’Sullivan ML, Mukherjee D, Punal VM, Farsiu S, Kay JN. Anatomy and spatial organization of Muller glia in mouse retina. J Comp Neurol (2017) 525:1759–77. doi: 10.1002/cne.24153 27997986 PMC5542564

[106] Zahs KR, Newman EA. Asymmetric gap junctional coupling between glial cells in the rat retina. Glia (1997) 20:10–22. doi: 10.1002/(SICI)1098-1136(199705)20:1<10::AID-GLIA2>3.0.CO;2-9 9145301

[107] Tezel G, Chauhan BC, LeBlanc RP, Wax MB. Immunohistochemical assessment of the glial mitogen-activated protein kinase activation in glaucoma. Invest Ophthalmol Vis Sci (2003) 44:3025–33. doi: 10.1167/iovs.02-1136 12824248

[108] Inman DM, Horner PJ. Reactive nonproliferative gliosis predominates in a chronic mouse model of glaucoma. Glia (2007) 55:942–53. doi: 10.1002/glia.20516 17457855

[109] Wang L, Cioffi GA, Cull G, Dong J, Fortune B. Immunohistologic evidence for retinal glial cell changes in human glaucoma. Invest Ophthalmol Visual Sci (2002) 43:1088–94. 11923250

[110] Kanamori A, Nakamura M, Nakanishi Y, Yamada Y, Negi A. Long-term glial reactivity in rat retinas ipsilateral and contralateral to experimental glaucoma. Exp Eye Res (2005) 81:48–56. doi: 10.1016/j.exer.2005.01.012 15978254

[111] Gallego BI, Salazar JJ, de Hoz R, Rojas B, Ramirez AI, Salinas-Navarro M, IOP induces upregulation of GFAP and MHC-II and microglia reactivity in mice retina contralateral to experimental glaucoma. J Neuroinflamm (2012) 9:92. doi: 10.1186/1742-2094-9-92 PMC3410794 22583833

[112] Kerr NM, Johnson CS, Green CR, Danesh-Meyer HV. Gap junction protein connexin43 (GJA1) in the human glaucomatous optic nerve head and retina. J Clin Neurosci (2011) 18:102–8. doi: 10.1016/j.jocn.2010.06.002 20934339

[113] Heavner W, Pevny L. Eye development and retinogenesis. Cold Spring Harb Perspect Biol (2012) 4:a008391. doi: 10.1101/cshperspect.a008391 23071378 PMC3504437

[114] Mathieu E, Gupta N, Ahari A, Zhou X, Hanna J, Yücel YH. Evidence for cerebrospinal fluid entry into the optic nerve via a glymphatic pathway. Invest Opthalmol Visual Sci (2017) 58:4784–4791. doi: 10.1167/iovs.17-22290 28973323

[115] May CA, Lütjen-Drecoll E. Morphology of the murine optic nerve. Invest Ophthalmol Visual Sci (2002) 43:2206–12. 12091418

[116] Morrison JC, Johnson EC, Cepurna WO, Funk RHW. Microvasculature of the rat optic nerve head. Invest Ophthalmol Visual Sci (1999) 40:1702–9. 10393039

[117] Choi HJ, Sun D, Jakobs TC. Isolation of intact astrocytes from the optic nerve head of adult mice. Exp Eye Res (2015) 137:103–10. doi: 10.1016/j.exer.2015.06.014 26093274 PMC4523456

[118] Bernstein SL, Guo Y, Kerr C, Fawcett RJ, Stern JH, Temple S, The optic nerve lamina region is a neural progenitor cell niche. Proc Natl Acad Sci U.S.A (2020) 117:19287–98. doi: 10.1073/pnas.2001858117 32723825 PMC7431091

[119] Hubbard JA, Hsu MS, Seldin MM, Binder DK. Expression of the astrocyte water channel aquaporin-4 in the mouse brain. ASN Neuro (2015) 7:1759091415605486. doi: 10.1177/1759091415605486 26489685 PMC4623559

[120] Tezel G. Oxidative stress in glaucomatous neurodegeneration: mechanisms and consequences. Prog Retin Eye Res (2006) 25:490–513. doi: 10.1016/j.preteyeres.2006.07.003 16962364 PMC1820838

[121] Baxter PS, Hardingham GE. Adaptive regulation of the brain’s antioxidant defences by neurons and astrocytes. Free Radic Biol Med (2016) 100:147–52. doi: 10.1016/j.freeradbiomed.2016.06.027 27365123 PMC5145800

[122] Chen Y, Qin C, Huang J, Tang X, Liu C, Huang K, The role of astrocytes in oxidative stress of central nervous system: A mixed blessing. Cell Prolif (2020) 53: e12781. doi: 10.1111/cpr.12781 32035016 PMC7106951

[123] Bonvento G, Bolanos JP. Astrocyte-neuron metabolic cooperation shapes brain activity. Cell Metab (2021) 33:1546–64. doi: 10.1016/j.cmet.2021.07.006 34348099

[124] Bosco A, Breen KT, Anderson SR, Steele MR, Calkins DJ, Vetter ML. Glial coverage in the optic nerve expands in proportion to optic axon loss in chronic mouse glaucoma. Exp Eye Res (2016) 150:34–43. doi: 10.1016/j.exer.2016.01.014 26851485 PMC4972706

[125] Choi HJ, Sun D, Jakobs TC. Astrocytes in the optic nerve head express putative mechanosensitive channels. Mol Vis (2015) 21:749–66. 26236150 PMC4502055

[126] Hernandez MR, Miao H, Lukas T. Astrocytes in glaucomatous optic neuropathy. Glaucoma: Open Window to Neurodegeneration Neuroprotection (2008) pp:353–73. doi: 10.1016/S0079-6123(08)01125-4 18929121

[127] Rutigliani C, Tribble JR, Hagstrom A, Lardner E, Johannesson G, Stalhammar G, Widespread retina and optic nerve neuroinflammation in enucleated eyes from glaucoma patients. Acta Neuropathol Commun (2022) 10:118. doi: 10.1186/s40478-022-01427-3 35986368 PMC9392254

[128] Neufeld AH, Hernandez MR, Gonzalez M. Nitric oxide synthase in the human glaucomatous optic nerve head. Arch Ophthalmol (1997) 115:497–503. doi: 10.1001/archopht.1997.01100150499009 9109759

[129] Brown AM, Tekkok SB, Ransom BR. Glycogen regulation and functional role in mouse white matter. J Physiol (2003) 549:501–12. doi: 10.1113/jphysiol.2003.042416 12679378 PMC2342948

[130] Hildebrand C, Waxman SG. Postnatal differentiation of rat optic nerve fibers: electron microscopic observations on the development of nodes of Ranvier and axoglial relations. J Comp Neurol (1984) 224:25–37. doi: 10.1002/cne.902240103 6715578

[131] Yazdankhah M, Shang P, Ghosh S, Hose S, Liu H, Weiss J, Role of glia in optic nerve. Prog Retin Eye Res (2021) 81:100886. doi: 10.1016/j.preteyeres.2020.100886 32771538 PMC7865017

[132] Kalsi AS, Greenwood K, Wilkin G, Butt AM. Kir4.1 expression by astrocytes and oligodendrocytes in CNS white matter: a developmental study in the rat optic nerve. J Anat (2004) 204:475–85. doi: 10.1111/j.0021-8782.2004.00288.x 15198689 PMC1571318

[133] Ransom CB, Ransom BR, Sontheimer H. Activity-dependent extracellular K+ accumulation in rat optic nerve: the role of glial and axonal Na+ pumps. J Physiol (2000) 522 Pt 3:427–42. doi: 10.1111/j.1469-7793.2000.00427.x 10713967 PMC2269766

[134] Butt AM, Duncan A, Berry M. Astrocyte associations with nodes of Ranvier: ultrastructural analysis of HRP-filled astrocytes in the mouse optic nerve. J Neurocytol (1994) 23:486–99. doi: 10.1007/BF01184072 7983475

[135] Lee SH, Kim WT, Cornell-Bell AH, Sontheimer H. Astrocytes exhibit regional specificity in gap-junction coupling. Glia (1994) 11:315–25. doi: 10.1002/glia.440110404 7960035

[136] Chamberlain KA, Sheng ZH. Mechanisms for the maintenance and regulation of axonal energy supply. J Neurosci Res (2019) 97:897–913. doi: 10.1002/jnr.24411 30883896 PMC6565461

[137] Johri A, Beal MF. Mitochondrial dysfunction in neurodegenerative diseases. J Pharmacol Exp Ther (2012) 342:619–30. doi: 10.1124/jpet.112.192138 22700435 PMC3422529

[138] Karbowski M, Neutzner A. Neurodegeneration as a consequence of failed mitochondrial maintenance. Acta Neuropathol (2012) 123:157–71. doi: 10.1007/s00401-011-0921-0 22143516

[139] Golpich M, Amini E, Mohamed Z, Azman Ali R, Mohamed Ibrahim N, Ahmadiani A. Mitochondrial dysfunction and biogenesis in neurodegenerative diseases: pathogenesis and treatment. CNS Neurosci Ther (2017) 23:5–22. doi: 10.1111/cns.12655 27873462 PMC6492703

[140] Lin MT, Beal MF. Mitochondrial dysfunction and oxidative stress in neurodegenerative diseases. Nature (2006) 443:787–95. doi: 10.1038/nature05292 17051205

[141] Yu-Wai-Man P, Votruba M, Burte F, La Morgia C, Barboni P, Carelli V. A neurodegenerative perspective on mitochondrial optic neuropathies. Acta Neuropathol (2016) 132:789–806. doi: 10.1007/s00401-016-1625-2 27696015 PMC5106504

[142] Carelli V, Ross-Cisneros FN, Sadun AA. Mitochondrial dysfunction as a cause of optic neuropathies. Prog Retin Eye Res (2004) 23:53–89. doi: 10.1016/j.preteyeres.2003.10.003 14766317

[143] Minckler DS, McLean IW, Tso MOM. Distribution of axonal and glial elements in the rhesus optic nerve head studied by electron microscopy. Am J Ophthalmol (1976) 82:179–87. doi: 10.1016/0002-9394(76)90416-5 821348

[144] Andrews RM, Griffiths PG, Johnson MA, Turnbull DM. Histochemical localisation of mitochondrial enzyme activity in human optic nerve and retina. Br J Ophthalmol (1999) 83:231–5. doi: 10.1136/bjo.83.2.231 10396204 PMC1722931

[145] Ashrafi G, Schwarz TL. The pathways of mitophagy for quality control and clearance of mitochondria. Cell Death Differ (2013) 20:31–42. doi: 10.1038/cdd.2012.81 22743996 PMC3524633

[146] Morizawa YM, Hirayama Y, Ohno N, Shibata S, Shigetomi E, Sui Y, Reactive astrocytes function as phagocytes after brain ischemia via ABCA1-mediated pathway. Nat Commun (2017) 8:28. doi: 10.1038/s41467-017-00037-1 28642575 PMC5481424

[147] Sirko S, Irmler M, Gascon S, Bek S, Schneider S, Dimou L, Astrocyte reactivity after brain injury-: The role of galectins 1 and 3. Glia (2015) 63:2340–61. doi: 10.1002/glia.22898 26250529 PMC5042059

[148] Stancic M, van Horssen J, Thijssen VL, Gabius HJ, van der Valk P, Hoekstra D, Increased expression of distinct galectins in multiple sclerosis lesions. Neuropathol Appl Neurobiol (2011) 37:654–71. doi: 10.1111/j.1365-2990.2011.01184.x 21501208

[149] Belmares R, Raychaudhuri U, Maansson S, Clark AF. Histological investigation of human glaucomatous eyes: Extracellular fibrotic changes and galectin 3 expression in the trabecular meshwork and optic nerve head. Clin Anat (2018) 31:1031–49. doi: 10.1002/ca.23263 30117188

[150] Coughlin L, Morrison RS, Horner PJ, Inman DM. Mitochondrial morphology differences and mitophagy deficit in murine glaucomatous optic nerve. Invest Ophthalmol Vis Sci (2015) 56:1437–46. doi: 10.1167/iovs.14-16126 25655803 PMC4347310

[151] Zhu Y, Pappas AC, Wang R, Seifert P, Sun D, Jakobs TC. Ultrastructural morphology of the optic nerve head in aged and glaucomatous mice. Invest Ophthalmol Vis Sci (2018) 59:3984–96. doi: 10.1167/iovs.18-23885 30098187 PMC6082327

[152] Dias MS, Luo X, Ribas VT, Petrs-Silva H, Koch JC. The role of axonal transport in glaucoma. Int J Mol Sci (2022) 23:3935. doi: 10.3390/ijms23073935 35409291 PMC8999615

[153] Quintero H, Shiga Y, Belforte N, Alarcon-Martinez L, El Hajji S, Villafranca-Baughman D, Restoration of mitochondria axonal transport by adaptor Disc1 supplementation prevents neurodegeneration and rescues visual function. Cell Rep (2022) 40:111324. doi: 10.1016/j.celrep.2022.111324 36103832

[154] Quigley HA, Addicks EM. Chronic experimental glaucoma in primates. II. Effect of extended intraocular pressure elevation on optic nerve head and axonal transport. Invest Ophthalmol Visual Sci (1980) 19:137–52. 6153173

[155] Quillen S, Schaub J, Quigley H, Pease M, Korneva A, Kimball E. Astrocyte responses to experimental glaucoma in mouse optic nerve head. PloS One (2020) 15: e0238104. doi: 10.1371/journal.pone.0238104 32822415 PMC7442264

[156] Loov C, Mitchell CH, Simonsson M, Erlandsson A. Slow degradation in phagocytic astrocytes can be enhanced by lysosomal acidification. Glia (2015) 63:1997–2009. doi: 10.1002/glia.22873 26095880 PMC6728804

[157] Morales I, Sanchez A, Puertas-Avendano R, Rodriguez-Sabate C, Perez-Barreto A, Rodriguez M. Neuroglial transmitophagy and Parkinson’s disease. Glia (2020) 68:2277–99. doi: 10.1002/glia.23839 32415886

[158] Krasemann S, Madore C, Cialic R, Baufeld C, Calcagno N, El Fatimy R, The TREM2-APOE pathway drives the transcriptional phenotype of dysfunctional microglia in neurodegenerative diseases. Immunity (2017) 47:566–581 e9. doi: 10.1016/j.immuni.2017.08.008 28930663 PMC5719893

[159] Boza-Serrano A, Ruiz R, Sanchez-Varo R, Garcia-Revilla J, Yang Y, Jimenez-Ferrer I, Galectin-3, a novel endogenous TREM2 ligand, detrimentally regulates inflammatory response in Alzheimer’s disease. Acta Neuropathol (2019) 138:251–73. doi: 10.1007/s00401-019-02013-z 31006066 PMC6660511

[160] Burguillos MA, Svensson M, Schulte T, Boza-Serrano A, Garcia-Quintanilla A, Kavanagh E, Microglia-secreted galectin-3 acts as a toll-like receptor 4 ligand and contributes to microglial activation. Cell Rep (2015) 10:1626–38. doi: 10.1016/j.celrep.2015.02.012 25753426

[161] Margeta MA, Yin Z, Madore C, Pitts KM, Letcher SM, Tang J, Apolipoprotein E4 impairs the response of neurodegenerative retinal microglia and prevents neuronal loss in glaucoma. Immunity (2022) 55:1627–1644 e7. doi: 10.1016/j.immuni.2022.07.014 35977543 PMC9488669

[162] Wei X, Cho KS, Thee EF, Jager MJ, Chen DF. Neuroinflammation and microglia in glaucoma: time for a paradigm shift. J Neurosci Res (2019) 97:70–6. doi: 10.1002/jnr.24256 29775216 PMC6239948

[163] Jiang S, Kametani M, Chen DF. Adaptive immunity: new aspects of pathogenesis underlying neurodegeneration in glaucoma and optic neuropathy. Front Immunol (2020) 11:65. doi: 10.3389/fimmu.2020.00065 32117239 PMC7031201

[164] Tezel G, Wax MB. The immune system and glaucoma. Curr Opin Ophthalmol (2004) 15:80–4. doi: 10.1097/00055735-200404000-00003 15021215

[165] Chung WS, Clarke LE, Wang GX, Stafford BK, Sher A, Chakraborty C, Astrocytes mediate synapse elimination through MEGF10 and MERTK pathways. Nature (2013) 504:394–400. doi: 10.1038/nature12776 24270812 PMC3969024

[166] Hayakawa K, Esposito E, Wang X, Terasaki Y, Liu Y, Xing C, Transfer of mitochondria from astrocytes to neurons after stroke. Nature (2016) 535:551–5. doi: 10.1038/nature18928 27466127 PMC4968589

[167] Williams PA, Harder JM, Foxworth NE, Cochran KE, Philip VM, Porciatti V, Vitamin B(3) modulates mitochondrial vulnerability and prevents glaucoma in aged mice. Science (2017) 355:756–60. doi: 10.1126/science.aal0092 28209901 PMC5408298

[168] Tribble JR, Otmani A, Sun S, Ellis SA, Cimaglia G, Vohra R, Nicotinamide provides neuroprotection in glaucoma by protecting against mitochondrial and metabolic dysfunction. Redox Biol (2021) 43:101988. doi: 10.1016/j.redox.2021.101988 33932867 PMC8103000

[169] Obel LF, Muller MS, Walls AB, Sickmann HM, Bak LK, Waagepetersen HS, Brain glycogen-new perspectives on its metabolic function and regulation at the subcellular level. Front Neuroenerget (2012) 4:3. doi: 10.3389/fnene.2012.00003 PMC3291878 22403540

[170] Waitt AE, Reed L, Ransom BR, Brown AM. Emerging roles for glycogen in the CNS. Front Mol Neurosci (2017) 10:73. doi: 10.3389/fnmol.2017.00073 28360839 PMC5352909

[171] Pellerin L, Magistretti PJ. Glutamate uptake into astrocytes stimulates aerobic glycolysis: a mechanism coupling neuronal activity to glucose utilization. Proc Natl Acad Sci U.S.A (1994) 91:10625–9. doi: 10.1073/pnas.91.22.10625 7938003 PMC45074

[172] Yellen G Fueling thought: Management of glycolysis and oxidative phosphorylation in neuronal metabolism. J Cell Biol (2018) 217:2235–46. doi: 10.1083/jcb.201803152 29752396 PMC6028533

[173] Oe Y, Baba O, Ashida H, Nakamura KC, Hirase H. Glycogen distribution in the microwave-fixed mouse brain reveals heterogeneous astrocytic patterns. Glia (2016) 64:1532–45. doi: 10.1002/glia.23020 27353480 PMC5094520

[174] Choi HB, Gordon GR, Zhou N, Tai C, Rungta RL, Martinez J, Metabolic communication between astrocytes and neurons via bicarbonate-responsive soluble adenylyl cyclase. Neuron (2012) 75:1094–104. doi: 10.1016/j.neuron.2012.08.032 22998876 PMC3630998

[175] Quigley HA. Neuronal death in glaucoma. Prog Retinal Eye Res (1999) 18:39–57. doi: 10.1016/S1350-9462(98)00014-7 9920498

[176] Jassim AH, Coughlin L, Harun-Or-Rashid M, Kang PT, Chen YR, Inman DM. Higher reliance on glycolysis limits glycolytic responsiveness in degenerating glaucomatous optic nerve. Mol Neurobiol (2019) 56:7097–112. doi: 10.1007/s12035-019-1576-4 30980229 PMC6728180

[177] Rouach N, Koulakoff A, Abudara V, Willecke K, Giaume C. Astroglial metabolic networks sustain hippocampal synaptic transmission. Science (2008) 322:1551–5. doi: 10.1126/science.1164022 19056987

[178] Danesh-Meyer HV, Zhang J, Acosta ML, Rupenthal ID, Green CR. Connexin43 in retinal injury and disease. Prog Retin Eye Res (2016) 51:41–68. doi: 10.1016/j.preteyeres.2015.09.004 26432657

[179] Mayorquin LC, Rodriguez AV, Sutachan JJ, Albarracin SL. Connexin-mediated functional and metabolic coupling between astrocytes and neurons. Front Mol Neurosci (2018) 11:118. doi: 10.3389/fnmol.2018.00118 29695954 PMC5905222

[180] Harun-Or-Rashid M, Pappenhagen N, Palmer PG, Smith MA, Gevorgyan V, Wilson GN, Structural and functional rescue of chronic metabolically stressed optic nerves through respiration. J Neurosci (2018) 38:5122–39. doi: 10.1523/JNEUROSCI.3652-17.2018 29760184 PMC5977447

[181] Renault F, Formstecher E, Callebaut I, Junier MP, Chneiweiss H. The multifunctional protein PEA-15 is involved in the control of apoptosis and cell cycle in astrocytes. Biochem Pharmacol (2003) 66:1581–8. doi: 10.1016/S0006-2952(03)00514-8 14555237

[182] Fiory F, Formisano P, Perruolo G, Beguinot F. Frontiers: PED/PEA-15, a multifunctional protein controlling cell survival and glucose metabolism. Am J Physiol Endocrinol Metab (2009) 297:E592–601. doi: 10.1152/ajpendo.00228.2009 19531639

[183] Exler RE, Guo X, Chan D, Livne-Bar I, Vicic N, Flanagan JG, Biomechanical insult switches PEA-15 activity to uncouple its anti-apoptotic function and promote erk mediated tissue remodeling. Exp Cell Res (2016) 340:283–94. doi: 10.1016/j.yexcr.2015.11.023 26615958

[184] Greig FH, Nixon GF. Phosphoprotein enriched in astrocytes (PEA)-15: a potential therapeutic target in multiple disease states. Pharmacol Ther (2014) 143:265–74. doi: 10.1016/j.pharmthera.2014.03.006 24657708 PMC4127788

[185] Bradbury EJ, Burnside ER. Moving beyond the glial scar for spinal cord repair. Nat Commun (2019) 10:3879. doi: 10.1038/s41467-019-11707-7 31462640 PMC6713740

[186] Liu Z, Chopp M. Astrocytes, therapeutic targets for neuroprotection and neurorestoration in ischemic stroke. Prog Neurobiol (2016) 144:103–20. doi: 10.1016/j.pneurobio.2015.09.008 26455456 PMC4826643

